# Personal Health Practices

**DOI:** 10.1186/1472-6874-4-S1-S4

**Published:** 2004-08-25

**Authors:** Heather Maclean, Keva Glynn, Zhenyuan Cao, Donna Ansara

**Affiliations:** 1 Centre for Research in Women's Health, University of Toronto and Sunnybrook Women's Hospital, 790 Bay St., 7th Floor, Toronto, ON, Canada; 2 Centre for Research in Women's Health, University of Toronto and Sunnybrook Women's Hospital, 790 Bay St., 7th Floor, Toronto, ON, Canada; 3 Centre for Chronic Disease Prevention and Control, Health Canada, 120 Colonnade Rd, Ottawa, ON, Canada; 4 Centre for Research in Women's Health, University of Toronto and Sunnybrook Women's Hospital, 790 Bay St., 7th Floor, Toronto, ON, Canada

## Abstract

**Health Issue:**

There are differences in health practices and self-rated health among different socio-demographic groups of women. The relationship between socio-demographic status and a) a range of health behaviours and b) a combination of multiple risk and multiple health promoting practices were examined. The relationship between self-rated health and health practices was also assessed.

**Key Findings:**

There were geographic differences in health practices with women in British Columbia having the highest odds of engaging in multiple health promoting practices, while women in Quebec had the lowest. Reports of engaging in multiple risk behaviours were most common in Ontario. Women from Ontario had the highest odds of reporting very good/excellent health and women from British Columbia had among the lowest odds.

The data supported a strong social gradient between an increase in income/education and healthy practices, especially those that are health promoting. However, women with higher education were more likely to be overweight and those with higher incomes were more likely to drink alcohol regularly.

Immigrant women were less likely to engage in multiple health risk practices compared to Canadian-born women. However, they were less likely to report very good/ excellent health than non- immigrants. While marriage appeared to have a generally protective effect on women's health practices, single women were more likely to be physically active and have a normal weight.

**Data Gaps and Recommendations:**

More sensitive indicators need to be developed to better understand possible reasons for the socioeconomic gradient. Data collection should focus on both rural and Aboriginal populations.

## Background

In recent years, differences in health outcomes by socio-economic position have been recognized as a persisting trend in public health. [1] A prominent hypothesis in the literature has been that the increased mortality risk associated with low levels of income and education is due to an increased prevalence of risky health practices, such as smoking, binge drinking and physical inactivity. [1] However, a large body of research and theory demonstrates that such practices develop from a complex interplay of factors, including income, education, gender, age, social support, cultural background and physical environment, which create a range of life contexts within which an individual's capacity to adopt healthy practices is either enhanced or constrained.

The health practices selected for discussion in this chapter are those that have been shown to have different patterns in men and women: eating practices, exercise, weight control (reflecting the links between weight and food intake and exercise patterns), smoking, alcohol consumption, use of pain medication, and use of complementary and alternative therapies. Given the fact that there has already been considerable analysis of the differences in health behaviours between men and women, this chapter focuses on the differences in health behaviours and self-rated health among different socio-demographic groups of women.

Health practices have been shown to have an impact on subjective views of health, including self-rated health and global quality of life, [2] findings that support the WHO's definition of health as "a state of complete physical, mental and social well-being and not merely the absence of disease." [3] Findings from longitudinal analyses have shown that self-perceived health is predictive of mortality, chronic disease incidence, recovery from illness, functional decline and the use of medical services. [2,4] Further, measures of self-rated health have been found to be valid tests with good test-retest reliability and predictive power. [5] Because health status data are not available, self-rated health is used as a proxy for health status in this report.

## Methods

This chapter considers the social context of women's health practices and self-reported health. A summary of the literature is followed by a new analysis of data from the Canadian Community Health Survey (CCHS), which addresses the following questions:

• What is the relation between women's socio-demographic status and their health practices?

• What is the relation between women's socio-demographic status and their multiple risk- and multiple health-promoting practices?

• What is the relation between women's self-rated health and their health practices?

A secondary analysis of data from the CCHS, Cycle 1.1 (2000–2001) was conducted. The CCHS is a national, cross-sectional survey that had a total of 125,574 respondents from 136 health regions across the country.

### Measures

Binge drinking is defined as the consumption of five or more alcoholic beverages on at least one occasion in the past 12 months.

Change to improve health indicates whether individuals have made a change in their lifestyle to improve their health in the past 12 months.

Consumption of fruits and vegetables is measured by asking respondents the total number of servings of fruits and vegetables they consume per day. Data are presented for those who consumed more than five servings and for those who consumed less than five servings per day.

#### Health practices

The first section under "Literature Review" defines the health practices examined in this report. Further details on these measures can be obtained from Statistics Canada's CCHS (2001) documentation.

Level of physical activity is measured by asking respondents whether or not they had engaged in various leisure-time physical activities in the previous three months (e.g. walking, swimming, gardening, golfing, weight training, jogging or running) as well as the frequency and duration of these activities. Estimates of the amount of energy expenditure were used to classify respondents as active, moderately active or inactive. Active respondents engaged in a sufficient amount of physical activity to achieve cardiovascular health benefits, and moderately active respondents experienced some health benefits but little cardiovascular benefit. Data are presented for those considered active and inactive.

#### Immigrants

Long-term immigrants – women who arrived in Canada 10 or more years ago. Recent immigrants – women who have lived in Canada for less than 10 years.

Multiple health-promoting practices is an index that identifies respondents who engaged in two or more positive health practices, including physical activity, consulting an alternative health care provider, making a change to improve health in the past 12 months, and consuming more than five servings of fruits and vegetables per day.

Multiple risk practices is an index that identifies respondents who engaged in two or more negative health practices – smoking, binge drinking, physical inactivity, use of pain relievers, and/or consuming less than five servings of fruits and vegetables per day. The practices selected for the index are factors known to affect women's and men's health.

Patterns of overweight are measured using the Canadian guidelines for body weight classification in adults. The level of health risk is determined by measuring body mass index (BMI). Overweight is defined as a BMI of 25 to 27.

Regular drinker is defined as someone who has consumed alcohol once a month or more frequently in the last 12 months.

Self-rated health, which is a subjective, global assessment of one's health, is rated on a 5-point scale ranging from poor to excellent. In this report, comparisons are made between those who rated their health as excellent or very good versus those who rated their health as good, fair or poor.

Smoking status identifies individuals who currently smoke on a daily or occasional basis (in contrast to never smokers and past smokers).

Use of complementary and alternative therapies is defined as consultation with a chiropractor or an alternative health provider, such as an acupuncturist, homeopath, reflexologist or massage therapist, about physical, emotional or mental health in the past 12 months.

Use of pain relievers in the past month refers to use of pain relievers such as Aspirin or Tylenol (including arthritis medicine and anti-inflammatories) in the past 30 days.

#### Other variables used

Age: categorized as 12–19, 20–44, 45–64, and 65+ years.

Education: categorized as high education – secondary school graduation or more; low education – less than secondary school graduation.

Household income: This is based on income adequacy, which takes into account the household income as well as the number of people in the household, divided into two categories. High income – middle or high-income adequacy (~ 80%); low income – lowest income adequacy (~10%).

Marital Status: Married – married and common law; Combined single – never married, separated, divorced and widowed.

Immigrant status: immigrant or non-immigrant.

Geographic regions: Ontario, British Columbia, Prairies (Alberta and Manitoba), Quebec, the Atlantic Provinces (Newfoundland and Labrador, Nova Scotia, P.E.I., New Brunswick), North (Northwest Territories, Yukon and Nunavut).

### Literature Review

The Effect of Social, Economic and Demographic Factors on Health Practices

### Gender and Health Practices

Recent national reports [6-8] indicate that men and women have distinctly different lifestyles; they differ not only in whether they adopt certain health-related habits but also in their concerns about, or attitudes towards, health. [6] On the positive side, women appear to be more attuned to health issues and thus are more likely to make healthy lifestyle choices. They tend to make healthy food choices (80% versus 63%) and are much more weight conscious than men (59% versus 41%). [6] As a result, they are much less likely to be overweight or obese than men (36% versus 48%). [6]

Except in the youngest age groups, fewer women than men smoke (25% versus 28%), and they are much more likely to abstain from alcohol or to drink in moderation (25% versus 50%). [6] Women are also more likely to use complementary and alternative medicines (18% versus 14%). [8] However, this does not hold true for all types of alternative care; for instance, men and women were equally likely to have seen a chiropractor. [6]

On the negative side, women are significantly less physically active than men (19% versus 25%) and use painkillers more regularly (70% versus 58%). [6]. In addition, certain health practices of young women show some disturbing trends: they are five times as likely as young men to be underweight, [8] and their smoking rates exceed those of young men, the only age group in which this is so. [9] Also of concern are the rising rates of binge drinking and risky sexual behaviour among young women, which are now comparable to those of young men. [8]

The following section provides a summary of what is known about the associations between socio-demographic factors, women's health practices and self-rated health. Data on what is known about men are presented where they illustrate a noteworthy difference between the sexes.

### Geographic Variation and Health Practices

In general, unhealthy practices among women tend to dominate in Quebec and the Atlantic provinces. Quebec and Prince Edward Island (P.E.I.) have the highest rates of smoking (32%), followed closely by Newfoundland and Labrador, and Nova Scotia (31% each). [9] Quebec has the highest reported rate of regular drinkers (57%). In contrast, British Columbia and Alberta have the highest reported rates of physical activity (27% and 26% respectively) and P.E.I. the lowest (14%). In terms of women taking action to improve health, reports are highest in Ontario and B.C., and lowest in Saskatchewan and Newfoundland and Labrador (39% versus 41%). Finally, reported consultations with alternative health care practitioners are highest in B.C. [9]

In light of the concentration of poor health practices in Quebec and the Atlantic provinces, it is interesting that Quebec has the highest rate of excellent/very good self-reported health (27% among men and women), followed closely by Newfoundland and Labrador (26%). Saskatchewan and Nova Scotia have the lowest reported rates of excellent/very good health (17% and 20% respectively). [7] Once again, these data suggest that the factors affecting self-rated health are complex and not well understood.

### Income and Education

It is well documented that women and men with lower socio-economic status (SES) are significantly more likely to lead a sedentary lifestyle, to have poorer dietary habits, to be overweight and to smoke cigarettes than women and men with higher SES; [9,10] as well, they are more resistant to changing their health practices. [10,11]

The literature shows that activity levels increase as incomes increase, a finding that is supported by the results of the three waves of the National Population Health Survey (NPHS). [6-8] According to NPHS data (1996–1997), 51% of women with the highest incomes are physically inactive as compared with 60% at the lowest income level. [8]

Patterns of alcohol use by socio-economic status are more complex. [12,13] Findings from the 1996–1997 NPHS indicate that rates of binge drinking are greatest among women in the highest income bracket (19%) as compared with those in the middle and lower ones (14% and 10% respectively). Women who are regular drinkers are also more likely to have higher incomes as well as to be older, single and have a higher level of education. [14] With respect to food intake, lower-income women are more likely than those with higher incomes to describe their eating habits as fair or poor and to express concerns about the cost of low-fat foods. [8]

Finally, women with higher household incomes are more likely (20%) than those at lower income levels (12%) to report using alternative health care. [6] Similar trends exist with regard to education.

### Age

In general, advancing age is associated with poorer health practices and lower perceptions of personal health. As women age they tend to gain weight and engage in less physical activity [15,16], and they are more likely to report fair/poor health if they have experienced unhealthy weight gain. [2,17,18] According to NPHS data, young women have the lowest rates of obesity (5%) and women aged 55 to 64 the highest rates (approximately 17%), a pattern that is consistent across all three waves of the NPHS survey. [6]

Smoking rates among young women (12–17 years of age) in Canada are a growing concern, particularly as they now exceed those of young men. Continuing a trend observed in 1994–1995, the rate of smoking among girls aged 12 to 14 (10%) and 15 to 17 (29%) has remained substantially higher than among young men of the same age (6% and 22% respectively). Among women, smoking rates are highest (approximately 32%) in the 18 to 54 age range and lowest (15%) in those 65 and older. [9] In the mid-age groups the percentage of women who use tobacco is approaching that of men, in part because men have quit at higher rates than women. Smoking among women in this age group is associated with lower income and education, heavier drinking and inactivity. [14]

Increasing alcohol use among young women has also been shown to be a growing trend. [19] Young women (20–24 years) are among the largest consumers and abusers of alcohol. In fact, the proportion of women aged 20 to 24 classified as regular drinkers (who consumed one drink or more per month) almost doubled from 1994–1995 to 1996–1997. [7,8] Among women over the age of 64, the prevalence of regular drinking continues to decline gradually. [8]

For women, the use of alternative care is most common in young to middle adulthood. Of those in the 25–44 and 45–64 age groups, 19% reported consulting an alternative practitioner in 1998–1999, as compared with approximately 11% for both 18- to 24-year-olds and those aged 65 and over. [20]

### Immigrant Status

According to data from the (1994–1995) National Population Health Survey (NPHS), vital statistics (1985–1987 and 1990–1992) and the General Social Survey (GSS), female immigrants (particularly recent immigrants from non-European, non-traditional source countries) experience better health status than women born in Canada. [21-23] This finding is supported by both Australian and U.S. studies showing that for almost every health status indicator and socio-demographic characteristic, female immigrants who have spent less than 10 years in their host country are healthier than long-term immigrants (more than 10 years) and the native-born population. [24-26] Data from the 1996–1997 NPHS indicate that recent female immigrants are less likely to be regular alcohol drinkers and smokers, and less likely to be overweight than Canadian-born women. [23,24] On the other hand, they are also less likely to engage in physical activity and more likely to have poor nutritional habits than their native-born counterparts. [27,28] However, only 5% of Asian-born immigrants are obese as opposed to 12% of Canadians. [7]

In contrast, another Canadian study [29] based on data from the GSS (1985 and 1991) found that the health status of female immigrants did not differ significantly from that of native-born Canadians, nor were there changes in self-reported health status over time.

Canadian studies show that male and female immigrant health practices change over time to resemble those of native-born Canadians. According to this research, new female immigrants smoke less, use less alcohol and are less likely to be obese than long-term immigrants. [21,30] A Canadian survey indicated that recent arrivals in Windsor consumed less alcohol than their Canadian-born counterparts, although alcohol use was more prevalent among recent immigrants with higher education and income than among recent immigrants with lower education and income. [31]

### Marital Status

The literature shows that being married or living in a common-law relationship has a mixed effect on health practices. [32-34] Partnered women consume less alcohol and have fewer alcohol-related problems than single women. [32] Marriage has also been shown to have a positive effect on the quality of women's diets. On the negative side, married women with young children were less likely to be physically active than their single counterparts. [33] Research on the association between marital status and BMI is limited. However, one study on body image showed that body dissatisfaction occurs at comparable levels among married and single individuals. [34]

### Effects of Social, Economic and Demographic Factors on Self-Rated Health

Both income and education show a distinct, independent, positive gradient with self-rated health. [2,35] In Canada, Shields and Shooshtari [2] found that women in lower-income households had higher odds of reporting fair/poor health and lower odds of reporting very good/excellent health than those in more affluent households. Similarly, women with a post-secondary degree had higher odds of reporting very good/excellent health than those with less education.

Age also shows a distinct, independent, positive gradient with self-rated health. [35,36] One study [36] demonstrates that health practices have a greater impact on the self-rating of younger age groups, whereas functional ability has a greater influence on the ratings of seniors.

In terms of self-rated health, Shields and Shooshtari [2] found that women who had never been married had higher odds of reporting fair/poor health than women who were currently married or who had previously been married.

Recent Canadian studies have reached conflicting conclusions with respect to immigrants' self-reported health status. One study [22] used the 1994–1995 NPHS database and found immigrants experienced better health status than individuals born in Canada. In contrast, a more recent study [37] using the same database found that immigrants were more likely than non-immigrants to report poor health status. Within the immigrant group, immigrants of European origin and long-term immigrants were more likely to report fair or poor health status than their non-European counterparts who had recently arrived in Canada. [38]

### Health practices and self-rated health

Less information is available on health practices and self-rated health than on demographic factors and self-rated health. Canadian women in all age groups are slightly less likely than men to report very good or excellent health. [2] Important factors affecting women's perceptions of fair/poor health are unhealthy weight gain and a reduction in physical activity, whereas for men smoking and alcohol consumption are more predictive of reports of poor health. [39,40] In comparison with men, social structural factors (e.g. income, employment and education) also play a more important role in determining the health of women. [39] When the impacts of changes in health practices over time are considered, women's self-rated health status is not affected by improved health practices, such as increased physical activity. [2] However, other changes – such as a negative change in physical status or psychosocial factors – were associated with a corresponding shift in self-rated health. [2] Clearly, an individual's assessment of his or her health is a complex process that requires further research.

## Results

### Provincial and Territorial Variations

**Figure 1:**
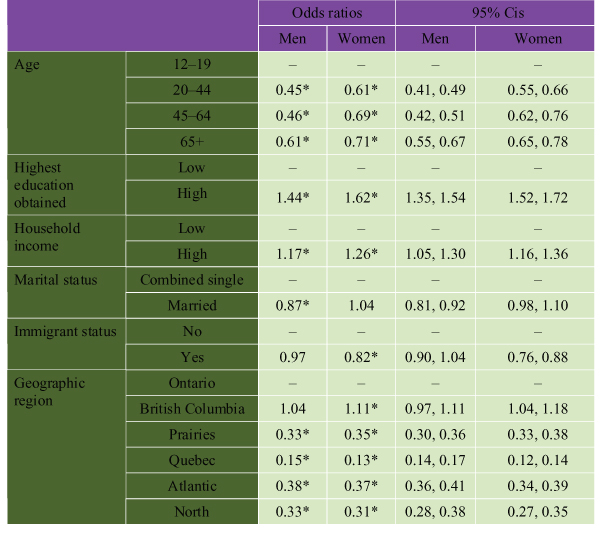
**Odds of Reporting Multiple Health-Promoting Practices^1,2 ^Controlling for Selected Demographic Factors**. Source: Statistics Canada, CCHS, 2000–2001. ^1^ Multiple health promoting practices is defined as having two or more of the following health promoting behaviours: being physically active, consulting an alternative health care provider, doing something in previous 12 months to improve health, consuming fruits and vegetables more than 5 times/serving per day. ^2^ All results were from Statistics Canada bootstrap programs. * Outcome is with multiple promoting practices.

**Figure 2:**
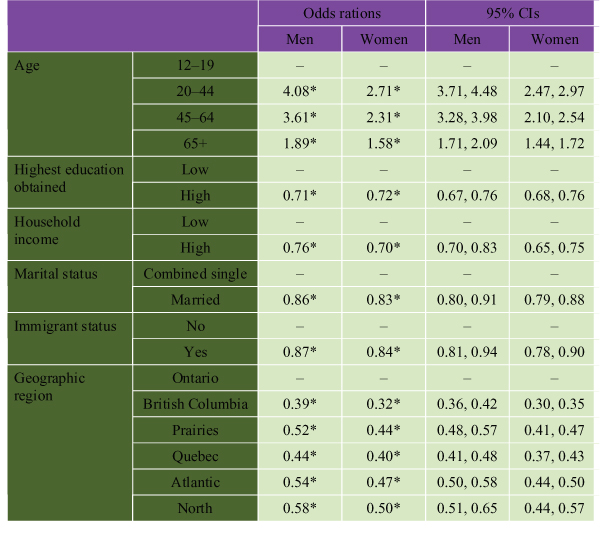
**Odds of Reporting Multiple Health Risk Factors^1,2 ^Controlling for Selected Demographic Variables. **Source: Statistics Canada, CCHS, 2000–2001. ^1^ Multiple health risk is defined as having two or more of the following health risk factors: smoking, using pain relievers, binge drinking, being physically inactive, consuming fruits and vegetables fewer than 5 times/serving per day. ^2^ All results were from Statistics Canada bootstrap programs. * Outcome is with multiple risk factors.

The geographic variation in women's health practices is depicted in Figures 1 and 2. Women in British Columbia had the highest odds of engaging in multiple health-promoting practices (odds ration [OR] 1.11, confidence interval [CI] 1.04, 1.18). In contrast, women in Quebec had the lowest odds of engaging in multiple health-promoting practices (OR 0.13, CI 0.12, 0.14).

Reports of multiple health risk factors were most common in Ontario. Of the remaining regions, women in the North and the Atlantic provinces were more likely to engage in multiple risk practices than women in Quebec, but their odds were approximately half those of women from Ontario (OR 0.50, CI 0.44, 0.57 and OR 0.47, CI 0.44, 0.50 respectively).

**Figure 3:**
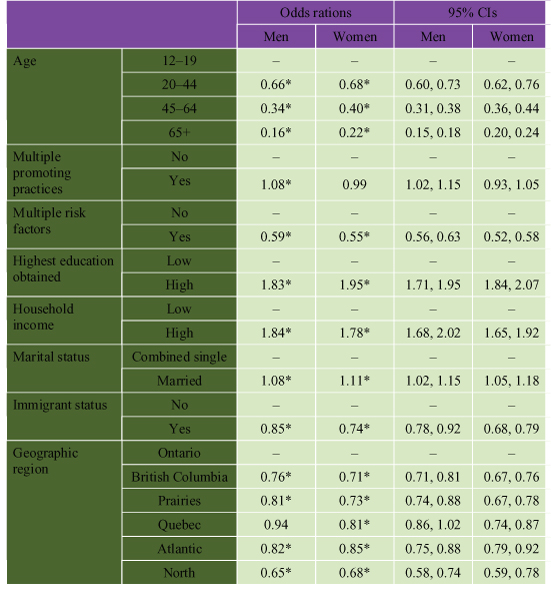
**Odds Of Reporting Excellent or Very Good Health Controlling for Multiple Health-Promoting Practices,^1 ^Multiple Health Risk Factors^2 ^and Other Selected Demographic Factors. **Source: Statistics Canada, CCHS, 2000–2001. ^1^ Multiple health promoting practices is defined as having two or more of the following health promoting behaviours: being physically active, consulting an alternative health care provider, doing something in previous 12 months to improve health, consuming fruits and   vegetables more than 5 times/serving per day. ^2^ Multiple health risk is defined as having two or more of the following health risk factors: smoking, using pain relievers, binge drinking, being physically inactive, consuming fruits and vegetables fewer than 5 times/serving per day. *  All results were from Statistics Canada bootstrap program

Our analysis of self-rated health by region revealed that when compared with women from Ontario, women from the other geographic regions had lower odds of reporting very good/excellent self-rated health (see Figure 3). Of the remaining regions, women from the Atlantic provinces were more likely than those from British Columbia, the Prairies, or the North to report very good/excellent health (OR 0.85, CI 0.79, 0.92).

### Income and Education

**Figure 4:**
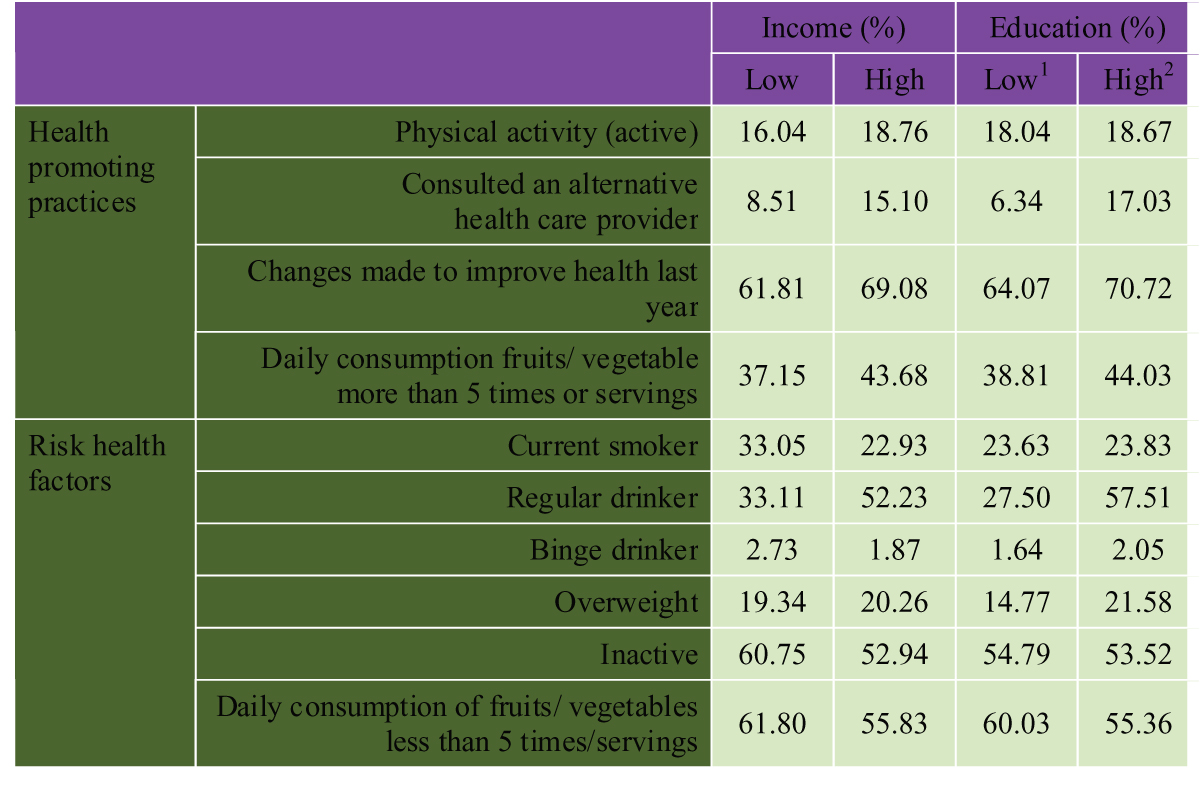
**Income/Education and Health Practices (not age-adjusted). **Source: Statistics Canada, CCHS, 2000–2001. ^1^Low education: less than secondary school graduate. ^2^High education: at least secondary school graduate.

Our findings support prior research suggesting a strong social gradient between an increase in income and education and healthy practices (see Figure 4). The trend was most apparent with respect to health-promoting practices. For example, 15.1% of women with higher incomes versus 8.5% of those with lower incomes consulted an alternative health care provider. Higher-income earners made changes to improve their health (69.1% versus 61.8%), they consumed more than five servings of fruits and vegetables (43.7% versus 37.2%) and were more physically active (18.76% versus 16.04%). Similarly, the likelihood of women with high levels of education compared to those with a low education level consulting an alternative health care provider was 17.0% versus 6.3%, making changes to improve their health was 70.7% versus 64.1%, and eating more than five servings of fruits and vegetables was 44.0% versus 38.8%.

Our findings in terms of the association between health risk factors, income and education followed a less distinct trend. The likelihood of higher income earners compared to low income earners smoking was 22.9% versus 33.1%, engaging in binge drinking was 1.9% versus 2.7%, and being inactive was 52.9% versus 60.1%. On the other hand, women with higher incomes were just as likely as those with lower incomes to be overweight (20.3% versus 19.3%) and the proportion of regular drinking was 52.2% versus 33.1%.

Smoking, physical activity, binge drinking and weight control displayed a different relation to income than to education. There was no difference in the proportion of current smokers between women with lower and higher levels of education (23.6% versus 23.8%), nor was there a difference between level of education and physical activity (low 18.0% versus high 18.7%). In addition, 2.1% of women with high levels of education reported binge drinking versus 1.6% of their less educated counterparts. Finally, 21.6% women with more education were overweight versus 14.8% of those with less education.

As shown elsewhere, [2] income and educational levels showed a strong, positive social gradient with self-rated health (see Figure 3).

### Age

**Figure 5:**
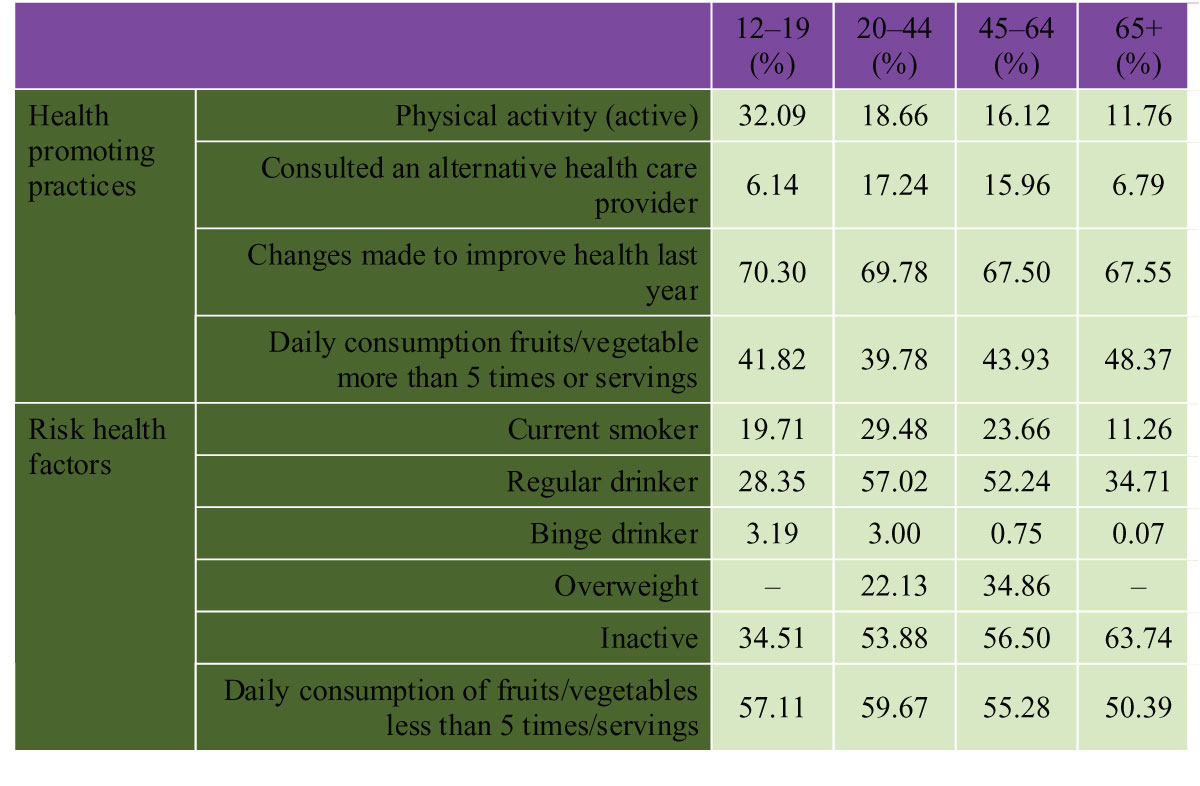
**Age and Health Practices (not age-adjusted) **Source: Statistics Canada, CCHS, 2000–2001

With the exception of physical activity, the analysis did not reveal a distinct trend between age and health-promoting practices (see Figure 5). There was little difference between the three age groups with respect to the likelihood of making changes to improve health, and likewise little difference in terms of fruit and vegetable consumption, although 48.4% of those 65 years of age and over consumed more than five servings of fruits and vegetables daily as compared with 39.8% of those aged 20 to 44. Of women 20 to 44 years of age, 17.2% consulted alternative health care providers, while of women 45 to 64 years of age, 16.0% did so.

Health risk activities were most likely among women 20 to 44 years of age and 45 to 64 years of age. The proportion smoking was 29.5% and 23.66% respectively, and regular drinking was 57.0% and 27.50% respectively. The proportion engaging in binge drinking was 3.0% among women aged 20 to 44 and 3.2% among those aged 12 to 19.

Consistent with other findings, [2] the current results indicate that reports of very good/excellent health were higher among younger women (see Figure 3).

### Immigrant Status

**Figure 6:**
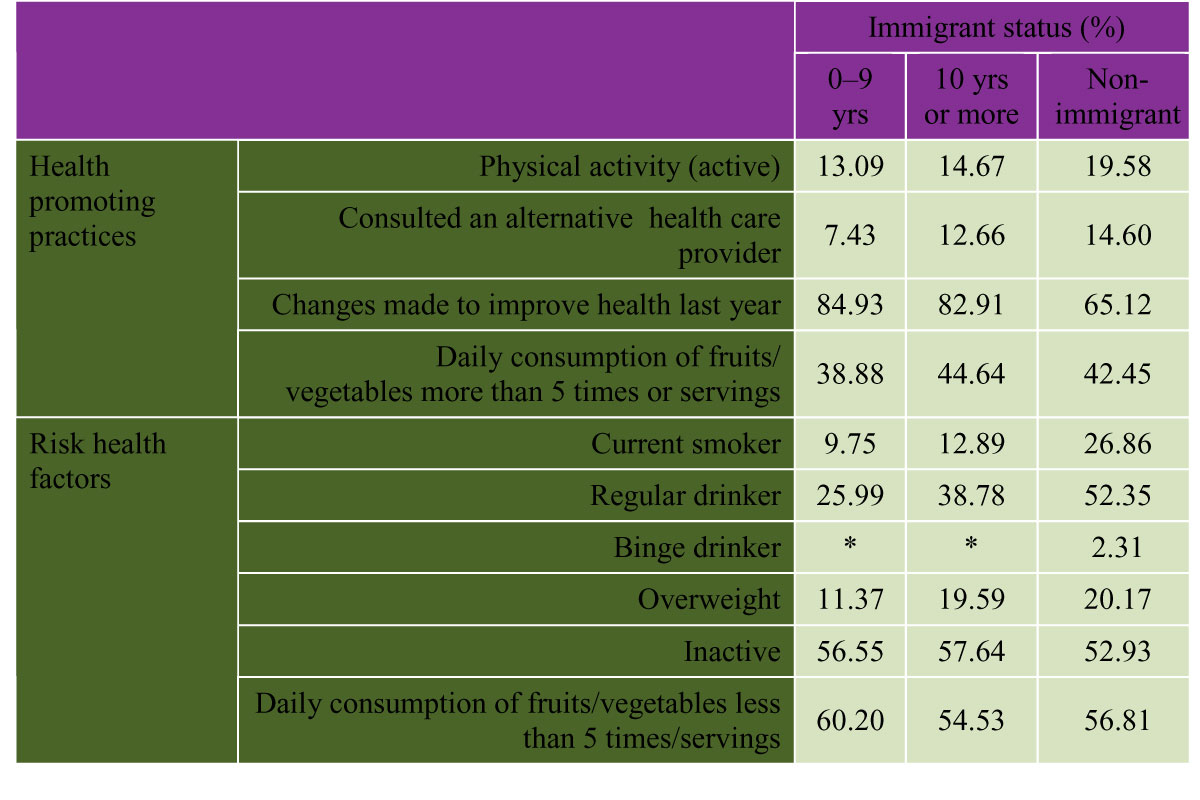
**Immigrant Status and Health Practices (not age-adjusted) **Source: Statistics Canada, CCHS, 2000–2001. * Insufficient sample size to report

Findings in this area were mixed (see Figure 6). The rates of smoking, regular drinking and multiple risk practices among non-immigrants reinforced previous findings. [21,22] However, the proportion of Canadian-born women who were physically active was 19.6% as compared with 14.7% among long-term immigrants, and the proportion consulting alternative health care providers was 14.6% versus 12.7%.

Prior research [21-29] suggests that the longer individuals stay in their host country, the more convergent their health practices become with those of their native-born counterparts. However, the current results in this regard were inconsistent. Although there was little difference between Canadian-born women and long-term immigrant Canadian women with respect to consumption of fruits and vegetables (42.5% versus 44.64% respectively) and being overweight (20.2% versus 19.6% respectively), Canadian-born women reported rates of smoking of 26.9% versus 12.9% reported by long-term immigrant Canadian women, and regular drinking of 52.4% versus 38.8% by long-term immigrant women. The analysis revealed that immigrants (both long-term and more recent) were less likely to engage in multiple risk health practices than their Canadian-born counterparts. The proportions of recent immigrants, long-term immigrants and non-immigrants who made changes to improve their health in the previous year were 84.9%, 82.9% and 65.1% respectively.

Finally, immigrants (both long-term and more recent) were less likely than non-immigrants to report very good/excellent health (OR 0.74, CI 0.68, 0.79) (see Figure 3).

### Marital Status

**Figure 7:**
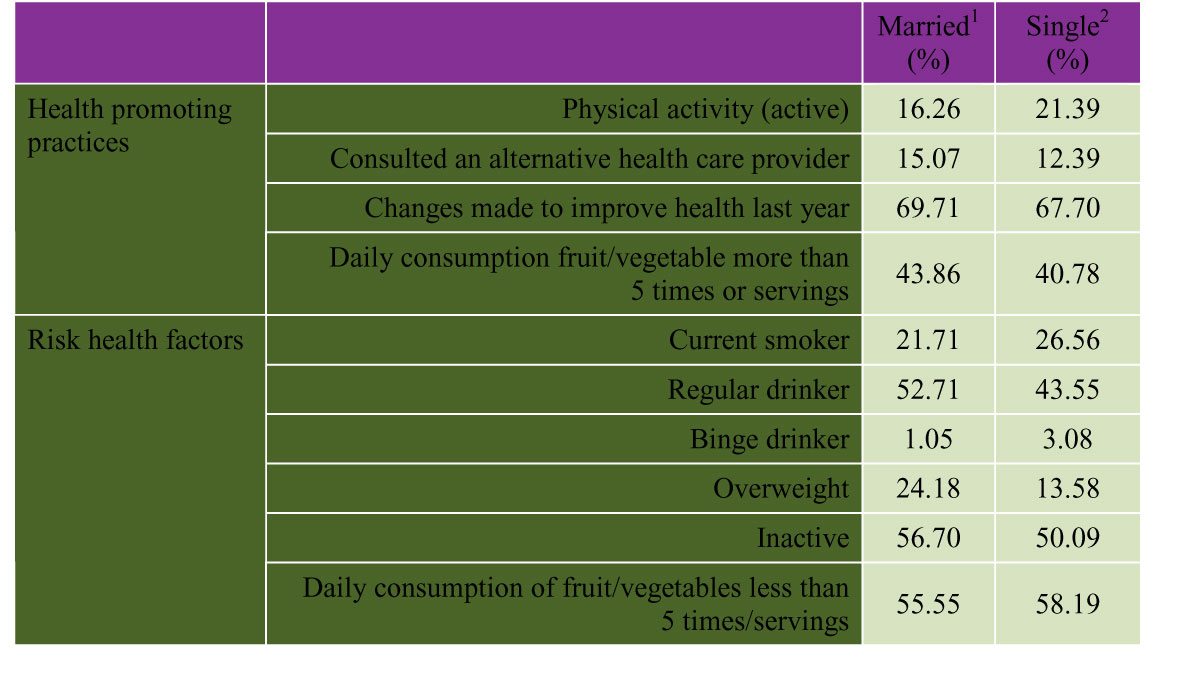
**Marital Status and Health Practices (not age-adjusted). **Source: Statistics Canada, CCHS, 2000–2001. ^1^ Married status includes married and common-law. ^2^ Single status includes widowed, separated and divorced.

The findings presented here partially support the claim that marriage has a protective effect on women's health practices (see Figure 7). The proportion of married/partnered women compared with un-partnered women who reported smoking was 21.7% versus 26.6% and for binge drinking was 1.1% versus 3.1%. With respect to the consumption of more than five servings of fruits and vegetables daily and consulting an alternative health care provider the proportions for married/partnered women compared with un-partnered women were 43.9% versus 40.8%, and 15.1% versus 12.4% respectively. On the other hand, the proportion of married/partnered women who reported being physically active was 16.3% versus 21.4% for their single counterparts. Proportions reported for overweight were 24.2% and 13.6% for the partnered and un-partnered groups respectively. There was little difference between the two groups with respect to changes made to improve their health in the previous year (69.7% married versus 67.7% single).

Finally, married women had slightly higher odds of reporting very good/excellent health than their single counterparts (OR 1.11, CI 1.05, 1.18) (see Figure 3).

## Discussion

### Geographic Variation

The results of the analysis of geographic variation in health practices were largely consistent with previous research. The healthiest practices were found in British Columbia, where rates of physical activity may be higher partly because of more clement weather and a distinct culture that values physical exercise. Other practices more common in British Columbia, such as attention to food selection and consultations with alternative health care practitioners, are consistent with B.C.'s position as one of the top three wealthy provinces in the country. [41] On the other hand, women from Quebec, a province of comparable wealth, had the lowest odds of engaging in multiple health-promoting practices. The marked difference in behaviours, despite similar provincial economic circumstances, suggests that health practices may be more closely associated with historic differences in cultural values than with socio-economic status. [42]

The finding that, compared with Ontario, all other regions in Canada had lower odds of engaging in risky health practices was surprising given previous research showing riskier health practices in Quebec, the Atlantic provinces and the North. [7,8] This discrepancy may be due, in part, to the indices for multiple health risk used in the report.

Despite engaging in riskier health practices, women from Ontario had higher odds than women from other geographic regions of reporting very good/excellent health. Also, the relatively high odds of very good/excellent health of women from the Atlantic provinces conflict with previous research showing a strong socio-economic gradient with self-rated health. [2] The discrepancy between poor health behaviours and positive self-perceptions of health in Quebec, as well as the surprising results from the Atlantic provinces, raises the possibility that other components beyond traditional socio-demographic factors, such as social, cultural, political and environmental contexts, may affect perceptions of health. [43,44]

### Income and Education

Although the finding of a social gradient between healthier practices and increased income and education supports previous research, the trend was more consistent for health-promoting than for health risk practices. [1,7,10] Previous research indicating that health risk practices are more common among those with lower incomes and less education was not consistently supported by our findings, although income appears to have more of a protective effect on health practices than education. Indeed, women with higher levels of education appear to be as likely, and in some instances (binge drinking, overweight) more likely, to engage in risky health practices. In particular, the present finding that being overweight is more common among women with higher education conflicts with previous research. However, these results need to be interpreted with caution, as the data in Figure 6 were not age-adjusted and this could be causing age confounding. The difference could also be a result of variation in the definition of the education variable rather than a new finding. Clearly, more research is needed to explore the complex interplay of income and education with other social factors, including social support and the physical environment. In addition, an increased awareness among health professionals of the cluster of risky health practices more common among higher income earners and women with more education is also of importance.

### Age

Results in this area did not support previous findings of a substantial trend towards poorer health practices (particularly smoking) among very young (12- to 19-year-old) women. [7,43] Indeed, Figure 2 shows that all older age groups are at higher risk for multiple risk factors than 12- to 19-year-olds. However, the results were limited by the fact that the CCHS data are cross-sectional, and therefore changes over time within the age groups were not evident in our study. Further, because the samples were small the parameters used to define the age cohorts were necessarily broad. Another possibility is a "cohort effect" in which a generation of women who engaged in risky health practices is now in the 20 to 44 age grouping, resulting in a clustering of poor health practices in this age group. Finally, previous analysis [8] compared smoking and drinking rates between young women and young men rather than comparing age groups of women. More research is needed on the health practices of young women to determine the age groups at greatest risk.

Despite healthier behaviours, older women were less likely to report very good/excellent health. It is thought that the higher incidence of chronic health conditions among older women may contribute to their lower ratings of health.

### Immigrant Status

Comprehensive explanations for the differences between long-term immigrants' and non-immigrants' health practices are complex. Some of the differences may be due to income disparities between immigrant and non-immigrant women. [8] Increased physical activity, attention to diet and consultations with alternative health providers are strongly associated with higher incomes and are also significantly more prevalent among non-immigrants. [8] Age may also be a contributing factor, in that immigrant women tend to be older than their Canadian-born counterparts, and advancing age is associated with a more sedentary lifestyle. [8] Finally, the consistently higher reports of smoking by non-immigrants may have a cultural as well as an economic component.

### Marital Status

The results confirm prior research on the protective effects of partnership. [32] Given that partnered women tend to have higher household incomes, their higher rates of consultation with alternative health care providers and attention to diet are consistent with the association between income level and these practices.

The slightly greater likelihood that married women will report very good/excellent health fits with numerous studies indicating improved mental and physical health with marriage. [44,45] Further research is required in this area to determine additional factors influencing the health practices of single women in relation to health status. Of particular interest is the impact of social values and community resources on women's ability and motivation to adopt self-care practices, and the subsequent impact on health status.

### Limitations to the Analysis

Because of the cross-sectional nature of the data in the CCHS it was not possible to draw conclusions about causal relations and outcomes. This limitation was particularly relevant with respect to the analysis of age-related differences in women's health behaviours, in that data on changes over time within the age groups would have strengthened the analysis.

A further limitation relates to how health practice variables should be operationalized. The smoking variable determined current smoking status. As a result of the substantial decrease in smokers in the past three decades (50% to 30%) it is possible that a large number of those who were not current smokers were actually former smokers. Grouping former smokers with non-smokers could result in misleading differences between socio-demographic groups of women. The "active" classification of exercise includes both high-intensity and moderate exercise, which could also lead to discrepancies in results between socio-demographic groups.

Finally, because of limited cell sizes, the data in the tables on income, education, immigrant status and marital status are not adjusted for age. As a result, age differences may confound the results, and the reader should interpret the findings with caution.

## Recommendations

### Policy Recommendations

1. This study has highlighted subgroups of women who demonstrate particularly poor health practices: women of low income, established Canadian immigrants (as compared with recent immigrants), and women living in Ontario and Quebec. It has also pointed to possible discrepancies between women's health practices and their self-rated health, particularly among young women and women from Quebec. On the basis of our findings, the following recommendations are made for future policy consideration.

2. Develop more sensitive indicators to capture other potential influences on women's health. Kawachi et al. [44] found that women experience higher rates of illness and death in those U.S. states that allow them lower levels of political participation and economic autonomy. Developing indices to measure the effects of broader influences on health, such as women's political participation, economic autonomy, employment and earnings, and reproductive rights, would provide important information with respect to women's health.

3. Develop the tools and resources necessary to conduct longitudinal studies on the personal health practices and health outcomes of female immigrants. The lack of data on immigrants' health practices limits the extent to which we can understand the underlying causes of the changes in those practices and the subsequent impact they may have on health outcomes.

4. Develop the tools and resources necessary to gather more data on the factors beyond traditional socio-demographic variables that may affect health practices and perceptions of health. In particular, more sensitive indicators are needed to help understand influences such as the education and income level of women's parents on their health practices, as well as the influence of cultural and geographic norms.

5. Address the lack of information on health practices of women in rural areas, and in particular in Nunavut, the Yukon and the Northwest Territories. Over 20% of women live in rural areas. [8] Women in rural areas are known to have substantially lower levels of income, education and employment. Given the strong association between these factors and poor health practices and self-rated health, the lack of data on Canada's rural areas, and particularly on women in the North, is disconcerting.

6. Address the lack of information available on Aboriginal women's health practices by ensuring their inclusion in national surveys. Given what is already known about the poor health practices and self-rated health of Aboriginal women, [7] there is a pressing need for more information on the health practices of this vulnerable subgroup.

7. Conduct further research to elucidate the contextual factors underlying regional differences in culture, values and behaviour as they relate to health and health practices.

8. Acknowledge the importance of socio-economic and cultural conditions and their influence on health practices. The results from this study support findings from previous analyses suggesting that socio-economic factors play a significant role in health practices. Therefore, in addition to focusing on individual health behaviours there is a need to look at policy directives that aim to decrease both socio-economic inequities and inequities between cultures.

9. Develop targeted health education programs promoting healthy individual behaviours for Aboriginal women, women with lower incomes and young women (20–44 years of age).

10. Conduct more research to understand the cultural reasons for the health practices of subgroups of women. Of particular concern are vulnerable subgroups, including young women (20 to 44 years) and women of low income.
